# Non-contrast peripheral angiography at 3T using QISS: improving venous suppression

**DOI:** 10.1186/1532-429X-16-S1-P168

**Published:** 2014-01-16

**Authors:** Shivraman Giri, Oisin Flanagan, Peter Speier, Ioannis Koktzoglou, Robert R Edelman

**Affiliations:** 1 Siemens Healthcare, Chicago, Illinois, USA; 2 Radiology, NorthShore University HealthSystem, Evanston, Illinois, USA; 3 Radiology, Feinberg School of Medicine, Northwestern University, Chicago, Illinois, USA; 4 Radiology, Pritzker School of Medicine, University of Chicago, Chicago, Illinois, USA; 5 Siemens Healthcare, Erlangen, Germany

## Background

Quiescent Interval Single Shot (QISS) has emerged as a robust technique for non-enhanced angiography of peripheral arteries (1). It has been clinically validated with contrast enhanced magnetic resonance angiography (CE-MRA) and digital subtraction angiography (DSA) (2,3). Both of these validation studies were performed at 1.5T field strength. Although the initial experience with QISS at 3T using similar imaging parameters as those at 1.5T were promising, venous suppression was inadequate in the thigh and pelvic regions of some patients (4,5). In this work, we present a strategy to improve venous signal suppression with the QISS sequence at 3T.

## Methods

The imaging parameters were similar to those reported earlier for QISS at 1.5T (2) with the following exception: the tracking saturation pulse for venous signal attenuation was replaced with an adiabatic inversion pulse (hyperbolic secant). This enables a more homogenous venous suppression at 3T than is possible with regular sinc pulse due to B1 inhomogeneity. The prototype sequence was tested in three volunteers in a 3T system (MAGNETOM Skyra, Siemens Healthcare).

## Results

**Figure 1:**
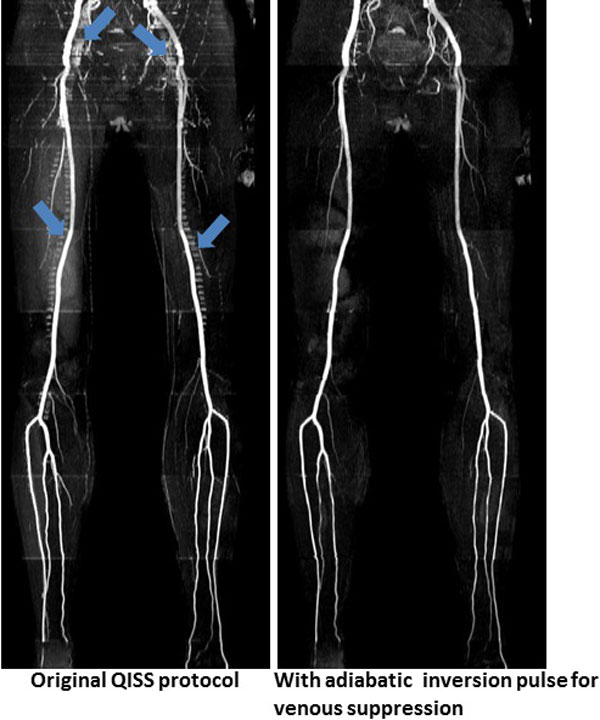
**Comparison of original QISS protocol (left) with the modified QISS (right)**. The arrows point to the undersirable vernous signal that has been nearly eliminated with the modified version of QISS.

The use of adiabatic inversion pulse instead of a sinc saturation pulse improved venous signal suppression, as shown in Figure 1, which also shows improved background signal suppression with the use of adiabatic inversion pulse. Improved suppression of veins and background permits better visualization of arteries. Similar results were observed in other subjects.

## Conclusions

Although QISS has shown robust performance at 1.5T, initial experience at 3T demonstrated suboptimal venous suppression. Although not established, this could be attributed to increased B1 inhomogeneity at 3T. We have demonstrated an approach to improve venous signal suppression at 3T using QISS. More patient studies are required and are underway to test the robustness and consistency of this approach.

## Funding

The primary author is a full time employee of Siemens Healthcare.

